# The changing pattern of COVID-19 in Nepal: A Global concern- A Narrative Review

**DOI:** 10.3126/nje.v10i2.29769

**Published:** 2020-06-30

**Authors:** Indrajit Banerjee, Jared Robinson, Abhishek Kashyap, Poornasha Mohabeer, Ananya Shukla, Alexandra Leclézio

**Affiliations:** 1 Associate Professor, Department of Pharmacology, Sir Seewoosagur Ramgoolam Medical College, Belle Rive, Mauritius; 2-6 Sir Seewoosagur Ramgoolam Medical College, Belle Rive, Mauritius

**Keywords:** Coronavirus, COVID-19, Mortality, Nepal, SARS-COV-2, Youth

## Abstract

This narrative review of the literature aims to assess the impact of COVID-19 on the younger age group in terms of the Global mortality of COVID-19 in comparison to Nepal. An extensive literature survey of English literature was conducted using Pubmed, Medline, Google Scholar, Embase, WHO Nepal Situation Updates on COVID-19, Situation update report, Ministry of Health and Population-Nepal from January 25, 2020 to June 20, 2020. According to the Ministry of Health and population of The Government of Nepal, as of June 20, 2020, out of a total of 8,605 laboratory confirmed cases reported to date, the pattern shows that most of the cases fell into the cohort of 21-30 years (37.72%), followed by 11-20 years (24.35 %), 31-40 years (21.97%) and 41-50 years (9.2%). To date Nepal has recorded a total of twenty-two deaths. At first evaluation these figures may not strike one as alarming, but on further investigation it is noted that the mean age is 42. 32 ± 19.632 SD years, and out of which male patients accounted for 77.3% and female accounted for 22.7%. The current situation of COVID-19 and how it develops in Nepal should be closely monitored and could be of international concern as it may be the early indicator of a changing pattern in COVID-19 infections. Nepal may therefore act as a global watch dog, due to the fact that the world could very possibly expose the younger age group under the notion that they are more resilient to the virus, when in reality that notion may be changing. This trend must be monitored and further investigated in order to establish the risk of the events unfolding in Nepal.

## Introduction

Coronavirus disease 2019 or (COVID-19) is caused by the novel beta coronavirus. SARS-CoV-2 [1] was a mere novelty and reached the odd newspaper headline prior to its identification as a global pandemic by the World Health Organisation in the second week of March 2020 [2]. In a minute duration of 6 months from the initial diagnosis of the first patient in Wuhan, China in December 2019 [3]. Till present day 20 June 2020 (8:09am CEST); there have been a staggering 8,465,085 confirmed cases with a global total of 454,258 deaths reported by the WHO. It has now been determined that COVID-19 originated from “Wuhan seafood market,”[4] a wet market privy to illegal wildlife trade. The virus hones in on the respiratory system and causes a host of respiratory symptoms [5, 6, 7]. The SARS-CoV-2 virus may also cause the precipitation of deadly complications [8]. According to the Ministry of Health and population, Government of Nepal on June 20, 2020 8,605 confirmed COVID positive cases have been reported [9]. According to the WHO, as of June 20, 2020, out of the top five countries which are worst hit by COVID-19, The United states of America is the global forerunner on the proverbial list. The US has a total of 2,172,212 COVID-19 positive cases with a loss of lives amounting to 118,205. This is followed by Brazil which has 978,142 positive cases with 47,748 Brazilians succumbing to the virus, Russia has 569,063 viral cases with 7,841 lives being lost. The fourth and fifth worst affected countries in the world are India which has 380,532 viral cases and deaths amounting to 12,573. The United Kingdom, which has 300,473 cases with deaths amounting to 42,288. (Data updated 08.09 am CEST, 20 June 2020) [3]. Among the five most adversely impacted countries, the deaths in the United Kingdom have been found to have the highest case fatality with 626 deaths per 1 million population having been reported. This is followed by the United States of America which has a death rate of 367, Brazil 231, Russia 55 and India 9 (per 1 million population).

Among all of the countries globally, the highest death rate was reported from San Marino with 1,238 deaths per 1 million population, this is followed by Belgium 837, Andorra 773, The United Kingdom 626, Spain 606, Italy 572 and Sweden with 500 deaths per 1 million population [3]. Whilst conducting a systematic review on the Epidemiology of COVID-19, a startling pattern was noted. To date age has been considered to be a predominant risk factor. Research conducted has reported that COVID 19 predominantly effects the elderly with concurrent comorbidities amounting to diseases in the spectrum of diabetes mellitus, cancer and cardiovascular diseases [10-12]. According to the latest WHO Nepal Situation Updates on COVID-19, the majority of the SARS-CoV-2 patients are male and the largest stake of these cases are in the cohort of 15-24 years, this is followed by the cohort of 25-34 years and 35-45 years respectively [13]. Therefore, this narrative review of the literature aims to assess the impact of COVID-19 on the younger age group in terms of the Global mortality of COVID-19 in comparison to Nepal.

## Methodology

### Literature searches

An extensive literature survey of English literature was conducted via the use of Pubmed, Medline, Google Scholar, Embase, WHO Nepal Situation Updates on COVID-19, Situation update report, The Ministry of Health and Population-Nepal from Jan 25, 2020 to June 20, 2020. A combination of Keywords was used “COVID 19”, OR “ Coronavirus”, OR “SARS-CoV-2” OR “Mortality” OR “Death” OR “Age ” OR “World” OR “Global” AND “Nepal”

### Inclusion/Exclusion Criteria

All of the research articles that were incorporated into the study were of the English language and was original research, published between January 25, 2020 and June 20, 2020; which focused on the age and the mortality of COVID-19 positive cases, and was focused on countries viz. Nepal and those most adversely affected by COVID 19 on the global scale. WHO Nepal Situation Updates on COVID-19, WHO Coronavirus Disease (COVID-19) Dashboard, Situation update report, Ministry of Health and Population-Nepal was also considered as there is a dearth of original studies in Nepal to date. Articles consisting of only the abstracts were rejected, the studies with the full text were included in the manuscript.

### Data Extraction

The data was extracted up until June 20, 2020 by two researchers (IB and JR) autonomously. The results of the numerous databases were initially searched for the suitable titles of various research papers. The designated titles were then screened for the abstracts as well as full texts and those that met eligibility requirements were considered for final selection. The extracted data comprised of the authors as well as the year of study, country of study, study design, sample size, sampling, the results and conclusion.

## Results

The literature searches generated a total of 781, 270 results, 6,925 results in Pubmed; 715,000 in Google scholar, 57,869 in Embase, Medline 1,335 results, WHO Nepal Situation Updates on COVID-19 9 results, Ministry of Health and Population-Nepal-132 results. 781,254 of the total 781, 270 results publications were excluded on initial screening. Therefore, only the relevant titles underwent a detailed assessment, and later on articles were excluded from the review process that finally resulted in eighteen relevant articles which were critically appraised and were included in this review.

### Current COVID-19 situation development in Nepal

Nepal comprises of 7 provinces viz. Bagmati Pradesh, Gandaki Pradesh, Province 1, Province 2, Province 5, Karnali Pradesh as well as Sudurpashchim Pradesh, and 77 districts. People in 73 of the 77 districts were found to be infected with the deadly SARS-CoV-2 coronavirus. The majority of these cases being imported from other countries [14]. According to the latest report published on 17th June 2020 by the WHO Nepal Situation Updates on COVID-19. The highest number of cases were reported from Province no. 2 (82%), Province no. 5 and Karnali province of Nepal. Interestingly, only 0.6 % of the cases were symptomatic at the time of their diagnosis. These cases have an overall case fatality of 0.28 % [13].

According to a report published by the Ministry of Health and population, Government of Nepal on June 20, 2020, there were 8,605 confirmed COVID positive cases which have been reported in Nepal. 7, 005 of which were in isolation, 1, 578 cases have been discharged. The Reverse transcription polymerase chain reaction (RT PCR) Test has been performed on 1, 69165 cases, the Rapid diagnostic test (RDT) was performed on 2, 52300 cases and currently 1, 02245 people are in Quarantine (updated on June 20, 2020) [15].

### Timeline of COVID-19 in Nepal

Patient zero in Nepal was documented on January 13, 2020 [16]. The case had a travel history and arrived from China. The case was a male individual who was 32 years in age and was a citizen of Nepal who was studying in Wuhan. He returned to Nepal for a winter vacation on January 9, 2020 [ 17, 18]. The throat swab was sent to Hongkong and was found to be COVID-19 positive on January 25, 2020 [19]. The second case of COVID-19 was reported 8 weeks later, the third case after two days, fourth positive case on March 27, 2020 and the fifth case was reported on March 28, 2020 [20].

The SARS-CoV-2 virus took approximately four months from early January 2020, to increase to 100 cases (May 7, 2020). In the next twenty days it was intensified to 1000 cases (1,042) on May 27, 2020, in the following 5 days it was intensified to 2000 cases (2,099) on June 02, 2020, in the next four days on June 6, 2020 it then escalated to 3000 cases (3,235), then to 4000 cases (4,035) on June 09, 2020, June 12, 2020 to 5000 cases (5,062), June 15 to 6000 positive COVID19 cases (6,211), June 17, to 7000 cases (7,177), to 8000 positive COVID-19 cases (8,274) on June 19, 2020 and was intensified to 8,605 on June 20, 2020 respectively [21]. The first death caused due to the deadly SARS-CoV-2 virus was reported on May 17, 2020 which was of a 29 year old female from the Sindhupalchowk district of Bagmati province who gave birth on May 08, 2020 (Fugure 1) [22].

### Age of the COVID-19 positive patients in Nepal Vs Global data

According to the latest WHO Nepal Situation Updates on COVID-19, The bulk of the patients belonged to the cohort of 15-24 (n=3025) 42.30% years, followed by 25-34 years (n=2043)28.57%, 35-45 years 16.05% (n=1148) respectively. The clinical Outcome of first 1759 patients in Nepal revealed that the majority (55%) of the patients were asymptomatic and were in isolation, 41.3% of the patients recovered from the disease, 1.4% were asymptomatic and were in Quarantine. 1.1% of the cases were symptomatic and were in isolation, death was reported to be among 1.1% of the COVID 19 patients [13].

**Figure 2:**
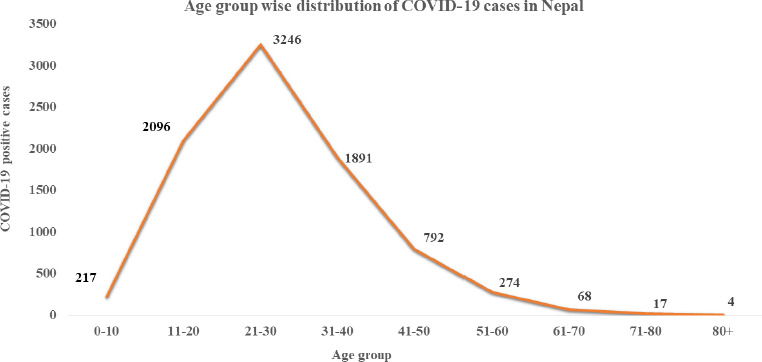
Age wise distribution of COVID 19 Patients in Nepal

Similar, findings were reported by the Ministry of Health and population, Government of Nepal on June 20, 2020, and the pattern remains the same. From a total of 8,605 laboratory confirmed cases reported to date, the pattern shows most of the cases stemmed from the cohort of 21-30 years n= 3246 (37.72), followed by 11-20 years n=2096 (24.35), 31-40 years n=1891 (21.97), 41-50 years n=792 (9.2), 51-60 years n= 274 (3.18), 61-70 years n= 68(0.79), 71-80 years n=17(0.19), 80+ years n=4 (0.046) (Figure 2) [15].

According to research performed by Joshi J et al. on the clinical profile of COVID 19 patients in the far Western province (Sudurpashchim Pradesh) of Nepal showed that the patients who were COVID-19 positive were generally males and that they were between 20-40 years in age [23]. This is highly varied from findings in China as according to a study conducted in China proved the average age of the COVID-19 cases to be 55·5 years [24]. In a study conducted in the USA by Aggarwal S in the Midwest states on 43 COVID positive patients found the mean age of the COVID-19 patients to be 65.5 years [25]. In another study from the USA, by Richardson S; it was reported that median age of infected individuals increased from 63 years of age to 65 years. [26]. According to a study done in Iran by Nikpouraghdam M, it has revealed that the majority of the cases were found in the older cohort and that COVID-19 was found to be more common in the cohort of 50 to 60 and in those of older age as well as those with concomitant comorbidities [27]. In Australia, of all the 295 cases the median age was found to be 47 years, with a major inclination to the cohorts of 50–59 and 60–69 years [28]. In Italy the median age of the cases was calculated to be 63 years of age [29] whereas in the United Kingdom the median age of 20133 patients admitted into acute centres across the United Kingdom amounted to 73 years in age [30]. In Brazil the age cohort of people who were 60+ years were most adversely impacted by COVID-19 [31]. In a study from the UK, One in five people over 80 years are covid-19(+) and have a statistically higher likelihood of requiring hospital admission as when equated to the 1% of people who are 30 years and younger who would require such medical intervention (Table 1) [32].

### Age of patient deaths due to COVID-19 in Nepal Vs Global data

On investigation it is noted that the mean age of the COVID-19 positive cases who died were 42. 32 ± 19.632 SD years, out of which male patients accounted for 77.3% and female patients accounted for 22.7% of the deaths in Nepal till June 20, 2020. According to the latest report published by WHO Nepal Situation Updates on COVID-19, on 17, June 2020 the highest death was reported in ≥ 55 years (5%) whereas infants and children only accounted for (1.18 %) in the age cohort of 0-4 years [13]. To date (June 20, 2020) Nepal has recorded a total of twenty two deaths [33].

On first evaluation these figures may not strike one as alarming, but on further investigation it is noted that; the initial death was of a female who was 29 years in age, the second death a 25 year old man, the third was a 41 year old male, the fourth a 70 year old man, the fifth a 56 year old man, the sixth case was a 35 year old man, the seventh case a 35 year old man, the eighth case a 2 years old girl, the ninth case a 76 year old man, the tenth case a 45 year old man, the eleventh case a 20 year male, the twelfth case a 58 year old male, the thirteen being a 55 year old male, the fourteen case a 58 year old male, the fifteenth case a 68 year old male, the sixteenth case a 57 year old male, the seventeenth case a 25 year old male, the eighteenth case a 5 year old boy, the nineteenth case a 36 year old man, the twentieth case being a 46 year old female, the twenty first and the twenty second case was 46 year old male and a 45 year old female respectively (Table 2).

According to the data from the WHO Nepal Situation Updates on death of the patients with known comorbid conditions only accounted for 12 of the deaths. Among the 7,173 COVID Positive cases in the age cohort of 75-84 years only 1 patient out of 8 (12.5%) died due to a comorbidity, this is followed by the age cohort of 65-74 years where 2 out of 31 patients (6.45 %) died due to a comorbidity, followed by the age cohort 55-64 years where 5 out of 118 patients (4.24 %) died due to known comorbid conditions [13]. Globally, in Germany the median age of the first consecutive 12 deaths was 73 years in age [34]. In Italy the majority of the deaths due to COVID-19 was found in the age group of more than 80 years. 3 out of the 21 deaths in Italy were from the age group of 60-69 years, 6 out of the 21 deaths were from the age group 70-79 years and 12 out of the 21 loss of lives were from the cohort of over 80 years in age [35]. A study conducted in Australia has reported that all the deaths that occurred were over 70 years in age [28].

In a study from the Republic of Korea, it was also found that 5 of the 27 deaths were in the cohort of 50-59 years, 6 of the 27 deaths were in the cohort of 60-69 years, 6 of the 27 deaths were in the cohort 70-79 years and 3 of the 27 deaths were 80+ years in age [36]. On the Diamond Princess ship the mean age on board was 58 years. Lives lost were 70+ years in age [37].

In a study from China the case fatality ratio was reported to be 1·38% (1·23–1·53), with substantially higher ratios in older age groups (0·32% [0·27–0·38] in those aged <60 years vs 6·4% [5·7–7·2] in those aged ≥60 years), up to 13·4% (11·2–15·9) in those aged 80 years or older.

These early estimates give an indication of the fatality ratio across the spectrum of COVID-19 disease and show a strong age gradient in risk of death (Table 1) [38].

## Discussion

Nepal has proven to be a substantial outlier in comparison to Global norms and standards. The initial pattern discovered in Nepal, presented to be a simple statistical outlier, however the data and results have yielded a concerning omen and potential threat to the global understanding of the transmission and established risk factors of the SARS-CoV-2 virus. The current situation developing in Nepal will be evaluated and discussed in comparison to the Global data.

### Age of COVID 19 Positive patients in Nepal:

According to research performed by Joshi J et al. on the clinical profile of COVID 19 patients in the far Western province (Sudurpashchim Pradesh) of Nepal it became evident that the majority of these COVID-19 positive cases were male; superimposed to this was the fact that these males were between 20-40 years in age [23]. According to a report published by the Ministry of Health and population, May 22, 2020, It was stated that bulk of the cases were of the ages between 21 to 30 years. This age group cohort therefore being in great deviation to international statistics and norms [39]. The current situation developing In Nepal is in stark contrast to Global data, this massive divide is becoming more evident as more data is being released from various regions across the globe.

### Global comparison of age:

On examination and analysis of the various age groups of SARS-CoV2 positive cases on the international spectrum, the degree to which Nepal strays from these norms becomes macroscopic. Research performed in Wuhan, China found that of 99 patients COVID19 patients that 86.6% were between the ages of ≤39- ≥70 years and the mean age was found to be 55.5 years [24]. An Iranian study further supports this by establishing the majority of their cases to be in the cohort between 50 to 60 years of age [27]. The WHO consider age to be a risk factor. Research performed by Aggarwal S et al in the United States of America [25] is supportive of the findings in Iran, China and of the WHO’s consensus as the mean age of those individuals infected with the virus was 65.5 years, this figure creating a gap of almost 30 years in difference as when compared to reports from Nepal. A report released from the COVID-19 National Incident Room Surveillance Team in Australia reveals of all the 295 cases the median age was found to be 47 years, with a major inclination to the cohorts of 50–59 and 60–69 years [28]. The Australian figures amounting rapidly and adversely to the 21 to 30 year old cohort displayed in Nepal. Research conducted in 12 hospitals of the New York city area discovered the median age from 5700 patients to be 63 years and that the age group cohorts were found to have an interquartile range of 52-75 years of age. It must be noted that on completion of the study that the median age of infected individuals increased from 63 years of age to 65 years;[26] this displaying and increase in age of infections as opposed to a decrease in age as depicted in Nepal. Italy depicted a median age of 63 years of age in a study conducted by Grasselli G [29]. Out of the 1591 cases in the study the age cohort centred around 56 to 70 years. The median age of the Italian study and that of the study conducted in New York were equal, therefore highlighting the differences between Nepal and the Global average. The median age of 20133 patients admitted into acute centres across the United Kingdom amounted to 73 years in age [30, 32]. Brazil’s COVID19 cases have shown an inclination and increased risk to the age cohort of above 60 years [31]. A similar trend is depicted in India, in a study conducted by Gupta N et al. reported that most of the cases are in the more aged cohort of over 50 years in age [40]. The global data of the world’s most adversely effected countries due to COVID19, form a tightly and closely related set of figures where the aged are predominantly effected. This global conglomerate frames the different picture being painted in Nepal in terms of the virus affecting the younger age cohorts.

### Deaths in Nepal due to COVID 19:

Out of the 22 deaths in Nepal the mean age of the COVID-19 positive cases who died were 42. 32 ± 19.632 SD years, and out of which male patients were 77.3% and female were 22.7%. This data and finding are extremely low as when compared to relative global data. The similarities between the global data and that of Nepal is the higher rates of deaths in male patients, however this is the only global trend that Nepal’s data subscribes to.

### Global Comparison of deaths:

On comparison of the various medians of age as well as age cohorts of deaths due to COVID-19 it is apparent that data from Nepal shows a much higher attrition rate in the lower age group than it does in the elderly.

Passengers on the Diamond Princess ship who contracted COVID19 and subsequently succumbed to the viral disease, were all above the age of 70 years and older [37]. This is in complete juxtaposition to the situation in Nepal. A study performed by Aggarwal S et al, in the United States of America found that those who succumbed to the virus were 70+ years in age, [25] this finding firmly cements and aligns with Global norms and further propels Nepal’s younger age of infections and death into the realms of Global importance and concern. The Korean society of infectious diseases have released data which indicates that 75% of deaths accounted for in Korea due to COVID19 were over the age of 50 years. 5 of the 27 deaths were in the cohort of 50-59 years, 6 of the 27 deaths were in the cohort of 60-69 years, 6 of the 27 deaths were in the cohort 70-79 years and 3 of the 27 deaths were 80+ years in age [36]. This data highlights the preponderance of deaths in COVID19 to be in the aged on the global scale, as opposed to the young in Nepal. An Italian study conducted by Rosella Porchedu et al, indicates that out of the study of 888 cases that the majority (12 out of 21 deaths) belonged to the cohort of those individuals being 80+ years in age [35]. Research conducted in Germany on the first 12 deaths due to COVID19, concluded that the median age of the first consecutive 12 deaths was 73 years in age [34]. The data is therefore unyielding in nature and it is evident that Nepal’s median death age is substantially lower than that of the Global data. The data from Nepal is unique and unlike any other reports. It is therefore vital and prudent for the Global law and policy makers to take into account and closely monitor the situation in Nepal before exposing the young and potentially putting their lives into danger.

## Conclusion

The current situation of COVID-19 and how it develops In Nepal should be closely monitored and could be of international concern as it may be the early indicator in a changing pattern of COVID-19 infections. Nepal may therefore act as a global watch dog, due to the fact that the world could very possibly expose the younger age group under the notion that they are more resilient to the virus, when in reality that notion may be changing. This trend must be monitored and further investigated in order to establish the risk of the events unfolding in Nepal.

## Figures and Tables

**Figure 1:**
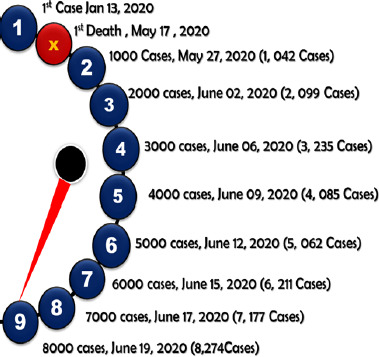
Timeline of COVID-19 cases in Nepal

**Table 1:** Age and mortality of the COVID-19 positive patients in Nepal Vs Global data

Author & year	Country	Study duration	Study design and population	Sample size	Sampling	Results/conclusion
**Joshi, 2020 [23]**	Nepal	27 March- 3rd April 2020-	Case Series	4	Convenience sampling	Age of COVID 19 patients from the Far Western Province ranged from 20 to 40 years.
**WHO, 2020 [13]**	Nepal	Jan 13, 2020-17th June 2020	Descriptive study	7152	Convenience sampling	Most patients were are of the cohort of 15-24 (n=3025) 42.30% years, followed by 25-34 years (n=2043)28.57%, 35-45 years 16.05% (n=1148) respectively..
**MOHP, [2020]** **[15]**	Nepal	Jan 13, 2020- June 20, 2020	Descriptive study	8,605	Convenience sampling	Out of 8,605 laboratory confirmed cases reported till date, the pattern shows most of the patients were in the age group 21-30 years n= 3246 (37.72), followed by 11-20 years n=2096 (24.35), 31-40 years n=1891 (21.97), 41-50 years n=792 (9.2), 51-60 years n= 274 (3.18), 61-70 years n= 68(0.79), 71-80 years n=17(0.19), 80+ years n=4 (0.046) cases who died were 42. 32 ± 19.632 SD years.
**Chen N [7]** **[2020]**	China	Jan 1 to Jan 20, 2020	Descriptive study	99	Convenience sampling	There was an inclination to men. The average age was calculated to 55·5 years. Older males with comorbid health conditions are more at risk.
**Nikpouraghdam M, [2020] [27]**	Iran	February 19, 2020 to April 15, 2020	Descriptive study	2968	Convenience sampling	Most cases were in the cohort of 50 to 60 years of older age and having comorbidities.
**Verity R [2020] [38]**	China	Jan 1, 2020- Feb 8, 2020.	Descriptive study	48	Convenience sampling	Case fatality ratio in China of 1·38% (1·23–1·53), with elevated ratios in greater aged groups (0·32% [0·27–0·38] in those <60 years vs 6·4% [5·7–7·2] in those aged ≥60 years), up to 13·4% (11·2–15·9) in those aged 80 years or older.
**Russell, 2020 [37]**	Diamond Princess ship	5 Feb -25 Feb 2020	Descriptive study	705	Convenience sampling	Mean age on board the ship was 58 years. Lives lost were 70+ years.
**Aggarwal, 2020 [25]**	USA	March 1 and April 4, 2020	Descriptive study	43	Convenience sampling	Average age of 65.5 years. Three patients were lost; all of which were 70+ in age.
**Korean society of infectious diseases, 2020 [36]**	Korea	January 19 to March 2, 2020	Descriptive study	2776	Convenience sampling	5 of the 27 deaths were in the cohort of 50-59 years, 6 of the 27 deaths were in the cohort of 60-69 years, 6 of the 27 deaths were in the cohort 70-79 years and 3 of the 27 deaths were 80+ years in age.
**Chea, 2020 [28]**	Australia	14 March 2020-unknown	Descriptive study	295	Convenience sampling	Of all the 295 cases the median age was found to be 47 years, with a major inclination to the cohorts of 50–59 and 60–69 years.
**Porcheddu, 2020 [35]**	Italy	February 20 to March 18, 2020	Descriptive study	888	Convenience sampling	3 out of the 21 deaths in Italy are of the cohort 60-69 years, 6 out of the 21 deaths are from the cohort 70-79 years and 12 of the 21 deaths were of individuals 80+ years in age.
**Richardson, 2020 [26]**	USA	March 1, 2020, to April 4, 2020	Descriptive study	5700	Convenience sampling	Median age of infected individuals increased from 63 years of age to 65 years .
**Grasselli, 2020** **[29]**	Italy	February 20 and March 18, 2020,	Descriptive study	1591	Convenience sampling	Median age of 63 years of age.
**Docherty, 2020 [30]**	UK	6 February, 2020 -Not known	Descriptive study	20133	Convenience sampling	The median age of 20133 patients admitted into Acute centres across the United Kingdom amounted to 73 years in age.
**Wichmann, 2020 [34]**	Germany	Not known	Descriptive study	12	Convenience sampling	The median age of the first consecutive 12 deaths were 73 years in age
**Mahase, 2020 [32]**	UK	Not known	Descriptive study	3665	Convenience sampling	Twenty five percent of people who are 80+ years in age who have the virus have a statistically higher likelihood of requiring hospital admission as when equated to the 1% of people who are 30 years and younger who would require such medical intervention.
**Leonardo Soares Bastos, 2020 [31]**	Brazil	Not known	Descriptive study	13008	Convenience sampling	In Brazil the age cohort of people who were 60+ years were most adversely impacted by COVID-19.
**Gupta, 2020 [40]**	India	February 15, 2020- March 19, 2020,	Multicentric descriptive, 41 sentinel sites	104	Convenience sampling	The most of the COVID 19 cases are in the more aged cohort of over 50 years in age.

**Table 2:** Demographic table of deaths reported in Nepal due to COVID-19

Death reported in Nepal	Age	Gender	District of Nepal	Provinces
1st death	29	Female	Sindhupalchowk	Bagmati
2nd death	25	Male	Banke	Province No. 5
3rd death	41	Male	Rupandehi	Province No. 5
4th death	70	Male	Bara	Province No. 2
5th death	56	Male	Lalitpur	Bagmati Province
6th death	35	Male	Arghakhanchi	Province No. 5
7th death	35	Male	Dailekh	Karnali Pradesh
8th death	02	Female	Bajura	Sudurpashchim Pradesh
9th death	76	Male	Dolakha	Bagmati Pradesh
10th death	45	Male	Palpa	Province No. 5
11th death	20	Male	Surkhet	Karnali Pradesh
12th death	58	Male	Dolpa	Karnali Pradesh
13th death	55	Female	Kailali	Sudurpashchim Pradesh
14th death	58	Male	Syangja	Gandaki Pradesh
15th death	68	Male	Chitwan	Bagmati Pradesh
16th death	57	Male	Arghakhanchi	Province No. 5
17 th death	25	Male	Parsa	Province No. 2
18 th death	05	Male	Bara	Province No. 2
19 th death	36	Male	Gulmi	Province No. 5
20^th^ death	46	Female	Banke	Province No. 5
21^st^ death	46	Male	Kailali	Sudurpashchim Pradesh
22^nd^ death	43	Female	Kailali	Sudurpashchim Pradesh
